# Combined Effects of Arsenic, Cadmium, and Mercury with Cardiovascular Disease Risk: Insights from the All of Us Research Program

**DOI:** 10.3390/ijerph22020239

**Published:** 2025-02-07

**Authors:** Oluwatobi L. Akinbode, Emmanuel Obeng-Gyasi

**Affiliations:** 1 Department of Built Environment, North Carolina A&T State University, Greensboro, NC 27411, USA; 2 Environmental Health and Disease Laboratory, North Carolina A&T State University, Greensboro, NC 27411, USA

**Keywords:** metals, metalloid, cardiovascular, mercury, arsenic, cadmium, mixtures

## Abstract

Background: Environmental exposures to heavy metals/metalloids such as arsenic, cadmium, and mercury have been implicated in adverse cardiovascular health outcomes. Using data from the All of Us research program, we investigated the associations between these metals/metalloids and six cardiovascular-related biomarkers: systolic blood pressure (SBP), HDL cholesterol, LDL cholesterol, C-reactive protein (CRP), total cholesterol, and triglycerides. Methods: This study explored the relationship between outcome cardiovascular variables (SBP, CRP, LDL, HDL, triglycerides, and total cholesterol) and predictor metal/metalloid variables (cadmium, mercury, and arsenic) among 136 participants (53.4 percent women). We initially conducted linear regression to determine the association between variables of interest. Bayesian Kernel Machine Regression (BKMR) analysis was subsequently performed to capture potential non-linear relationships, as well as interactions among metal/metalloid exposures. In the BKMR analysis, posterior inclusion probabilities (PIPs) quantified the contribution of each metal/metalloid to the outcomes, with higher PIP values indicating a greater likelihood of a specific exposure being a key predictor for a given cardiovascular biomarker. Within the BKMR framework, univariate, bivariate, and overall exposure–response analyses provided insights into the individual and combined effects of metal/metalloid exposures. These analyses identified the factors with the strongest associations and highlighted interactions between exposures. Results: In this study, the average age of male participants was 58.2 years, while female participants had an average age of 55.6 years. The study population included 104 individuals identifying as White (mean age: 57.5 years), 10 as Black or African American (mean age: 63.2 years), 7 as Hispanic (mean age: 48.2), 3 as Asian (mean age: 49.7 years), and 12 as Other race (mean age: 48.8 years). In our study, men exhibited higher levels of SBP, triglycerides, mercury, and arsenic, while women had higher levels of CRP, LDL cholesterol, HDL cholesterol, total cholesterol, and cadmium. Black people exhibited higher levels and greater variability in markers of cardiovascular risk and inflammation (e.g., blood pressure and CRP), Asians consistently showed the lowest levels across most biomarkers, while White people, Hispanics, and the “Other” group demonstrated moderate levels with some variability. In linear regression, we identified significant positive associations between mercury and HDL cholesterol, arsenic and triglycerides, and arsenic and total cholesterol. In BKMR analysis, PIP results revealed that mercury had the highest predictive contribution for SBP, HDL cholesterol, and triglycerides; cadmium for CRP; and arsenic for LDL and total cholesterol. Univariate and bivariate exposure–response analyses in BKMR demonstrated non-linear exposure–response patterns, including U-shaped and inverted U-shaped patterns for cadmium, particularly CRP and total cholesterol. Traditional linear regression techniques would have missed these patterns. Conclusion: Our study results highlight the influence of environmental metal/metalloid exposures on cardiovascular biomarkers, providing evidence of non-linear and interactive effects that warrant further investigation to understand their role in cardiovascular disease risk better.

## 1. Introduction

Cardiovascular disease (CVD) remains the leading cause of morbidity and mortality worldwide and represents a global public health challenge [1]. While traditional risk factors such as hypertension, dyslipidemia, smoking, and diabetes have been extensively studied, increasing attention is now being directed toward environmental exposures as significant contributors to CVD risk [2,3]. Among these, chronic exposure to toxic metals and metalloids—notably arsenic, cadmium, and mercury—has emerged as a critical yet underrecognized determinant of cardiovascular health.

Arsenic, a naturally occurring metalloid, and cadmium and mercury, classified as heavy metals, are widely distributed in the environment through both natural processes and anthropogenic activities [4]. Arsenic exposure primarily occurs through consuming contaminated drinking water, particularly in regions with elevated groundwater arsenic levels [5]. In contrast, cadmium and mercury are introduced into the human body predominantly via occupational exposure, cigarette smoking, and dietary intake, such as rice (a source of cadmium) and fish (a primary source of mercury) [6,7]. Given their biological and environmental persistence, these toxicants pose substantial public health risks, especially for vulnerable and marginalized populations.

Epidemiological and toxicological studies indicate that arsenic, cadmium, and mercury individually impair cardiovascular function through multiple mechanisms, including oxidative stress, chronic inflammation, endothelial dysfunction, and dysregulation of lipid metabolism [8,9,10]. However, real-world exposures are rarely limited to single toxicants; individuals are more commonly exposed to complex mixtures of metals and metalloids over their lifetime [11]. Despite this reality, the combined effects of these toxicants remain poorly understood, particularly in the context of cardiovascular disease risk. Emerging evidence suggests that interactions among these environmental toxicants can be synergistic or antagonistic, potentially amplifying or mitigating their overall health effects [12]. This complexity presents significant challenges for environmental health research and risk assessment.

This paper aims to address this critical knowledge gap by evaluating the combined effects of arsenic, cadmium, and mercury on cardiovascular disease risk. Leveraging data from the *All of Us* dataset and applying advanced statistical modeling techniques, we seek to provide a more comprehensive understanding of how exposure to these environmental toxicants influences cardiovascular outcomes. In doing so, this study underscores the urgent need for a mixture-based approach to assessing the cardiovascular risks associated with metal and metalloid exposures more effectively by accounting for interactions, non-linear relationships, and cumulative effects that traditional single-exposure models often overlook.

## 2. Materials and Methods

### 2.1. Data Source

This study utilized data from the *All of Us* research program, a nationwide initiative designed to collect comprehensive health-related information from a diverse participant pool across the United States [13]. The *All of Us* dataset integrates survey responses, physical measurements, electronic health records (EHRs), and genomic data, providing an unparalleled resource for health research. The *All of Us* researcher workbench, a secure platform equipped with advanced tools such as cohort builders and interactive notebooks, provides a means to access the data. Data used for this study included survey responses, physical measurements, and laboratory data on metal/metalloid exposure. Data for this analysis also included demographic and cardiovascular outcome measures.

### 2.2. Study Population

The study population consisted of 136 participants from the *All of Us* dataset based on the availability of complete data for mercury, which was chosen as the marker of interest for building the database as it allowed for the least missing data across variables. From this, levels of arsenic, cadmium, and six cardiovascular biomarkers were derived. Sociodemographic characteristics, including age, sex, and race/ethnicity, were analyzed. The ethnic makeup of study participants included White, Black, Hispanic, Asian, and Other.

### 2.3. Exposure Variables

The key exposure variables in this study were multimetal/metalloid data, which included arsenic, cadmium, and mercury levels. Metal/metalloid levels were measured in blood samples using standard laboratory procedures. Detailed information on the specific analytical methods, including quality control measures, can be found in the *All of Us* dataset documentation [13]. Arsenic, classified as a metalloid, is often found in contaminated drinking water and food sources. Cadmium exposure is associated with industrial environments and dietary intake, particularly rice and leafy greens. Mercury exposure is typically linked to fish consumption and environmental pollution. These metals and the metalloid were selected due to their established toxicological relevance and potential cardiovascular effects.

### 2.4. Cardiovascular Outcome Variables

Cardiovascular disease risk was assessed using six cardiovascular indicators: systolic blood pressure, low-density lipoprotein (LDL) cholesterol, high-density lipoprotein (HDL) cholesterol, C-reactive protein, total cholesterol, and triglycerides. These biomarkers were chosen based on their well-established association with cardiovascular health and disease risk, providing a robust framework for assessing cardiovascular outcomes.

The cardiovascular variables within this study derived from the following sources in the All of Us researcher workbench:

- Systolic Blood Pressure (SBP): Obtained from physical measurements recorded during clinical visits.
- LDL Cholesterol, HDL Cholesterol, Total Cholesterol, and Triglycerides: Measured from laboratory tests in the dataset. The All of Us program collects biosamples, including blood, used for various bioassays.
- C-Reactive Protein (CRP): Derived from laboratory analyses as part of inflammation-related biomarker assessments.

These data points are typically accessed through the *All of Us* researcher workbench, which integrates various data sources, including EHRs, physical measurements, and laboratory results. The biomarkers selected reflect objective measures recorded by healthcare professionals during participant evaluations, providing a comprehensive view of cardiovascular health indicators.

### 2.5. Data Analytics

#### 2.5.1. Descriptive Statistics and Correlation Analysis

The statistical analysis was conducted in four stages. First, descriptive statistics were used to summarize participant characteristics, metal and metalloid exposure levels, and cardiovascular indicators. Means and standard deviations were calculated for continuous and percentages for categorical variables. Visualizations, such as histograms and boxplots, were generated to assess the distributions of key variables and detect potential outliers.

Second, correlation analyses examined relationships between arsenic, cadmium, and mercury levels, and cardiovascular variables. Spearman’s rank correlation was used to account for potential non-linear associations. Correlation matrices and heatmaps were constructed to visualize these relationships.

We used the Spearman correlation method to measure the strength of the association among cardiovascular-related markers. This technique is particularly suitable because it measures a monotonic relationship, i.e., as one variable increases, the other either consistently increases or decreases. Compared to Pearson’s correlation, Spearman’s rank correlation is a non-parametric test that is less sensitive to outliers, as it uses rankings rather than the actual data values. This makes it especially effective when we know that outliers are present in the data. The Spearman rank correlation is mathematically represented as:

$$r_{s}=1-\frac{6\sum d_{i}^{2}}{n(n^{2}-1)}$$

where $d_{i}$ is the difference between the ranks of the i-th pair and n is the number of observations of the dataset.

The $r_{s}$ value ranges from −1 to +1. A positive value of $r_{s}$ indicates a positive relationship between two variables, while a negative value of $r_{s}$ indicates a negative relationship. When $r_{s}=0$, then there is no association between the two variables.

#### 2.5.2. Linear Regression Analysis

Multiple linear regressions were used to assess the combined effects of metals on cardiovascular disease makers. Adjustments for age, sex, and race were incorporated into all models.

In this study, we performed multiple linear regression analysis. The linear regression equation was as follows:

$$Y=\beta_{0}+\beta_{1}{Arsenic}_{i}+\beta_{2}{Cadmium}_{i}+\beta_{3}{Mercury}_{i}+\beta_{4}Age{+\beta_{5}Race+\beta_{6}Income+\varepsilon }_{i}$$

where Y represents different responses such as SBP, CRP, LDL, HDL, triglycerides, and total cholesterol and $\varepsilon_{i}$ ~ N(0, $\sigma^{2}$) is the random error term.

To ensure the validity of the linear regression models used in this study, a thorough assessment of model assumptions was conducted for cardiovascular outcomes (e.g., HDL, LDL, SBP, CRP, triglycerides, and total cholesterol) and metal exposures (arsenic, cadmium, and mercury). These checks confirmed that the assumptions of linear regression were met for all models.

The **normality of residuals** was evaluated using histograms with overlaid normal distribution curves, along with the Shapiro–Wilk test. Results indicated that residuals were approximately normally distributed for all models. **Homoscedasticity** was assessed through scatterplots of residuals versus fitted values, and the Breusch–Pagan test was used to confirm that residuals demonstrated constant variance across all levels of the predictors. Both visual and statistical assessments showed no evidence of heteroscedasticity.

To evaluate **linearity**, scatterplots with fitted regression lines were created for each cardiovascular outcome versus the predictors (arsenic, cadmium, and mercury). These plots demonstrated that the relationships between outcomes and predictors were approximately linear. **Multicollinearity** was assessed among the metal exposures (predictors) using Variance Inflation Factors (VIFs). All VIF values were below the commonly accepted threshold of 10, indicating no significant multicollinearity among the predictors. Lastly, the **independence of errors** was confirmed through the Durbin–Watson test, which showed no evidence of autocorrelation in the residuals.

These diagnostic checks demonstrated that the assumptions of linear regression were satisfied for all cardiovascular outcomes and metal exposures analyzed in this study, supporting the validity of the regression models.

#### 2.5.3. Bayesian Kernel Machine Regression

Finally, Bayesian Kernel Machine Regression (BKMR) was used to evaluate the combined effects of arsenic, cadmium, and mercury on cardiovascular outcomes.

The BKMR equation is shown below as:

$$Y_{i}=h\left(z_{i}\right)+x_{i}^{T}\beta +\varepsilon_{i},$$

where $Y_{i}$ represents a health outcome, h the flexible kernel function, and $z_{i}={\left(z_{i1}\ldots ,\;z_{iM}\right)}^{T}$ is a vector M exposure variable. The term $x_{i}$ includes a set of potential confounding variables, and $\varepsilon_{i}$ is an independent and identically distributed random error term assumed to follow a normal distribution, $\varepsilon_{i}\sim N(0,\;\sigma^{2})$.

The Bayesian Kernel Machine Regression (BKMR) model was applied to analyze the combined and interactive effects of arsenic, cadmium, and mercury exposures on cardiovascular outcomes. The model employs default prior distributions to facilitate flexible and robust analysis of non-linear relationships among exposures. Specifically, the regression coefficients (β) are assigned non-informative priors to allow the data to dominate the posterior estimates. Variance components are modeled using an inverse gamma prior (σ^−2^∼Gamma(a_σ_, b_σ_)) with hyperparameters a_σ_ = 0.001 and b_σ_ = 0.001.

The kernel-specific variance component (λ ≡ τσ^−2^) follows a gamma prior, and the inclusion probabilities for variable selection are governed by a beta prior. These default priors ensure the model’s flexibility while maintaining the ability to account for complex, non-linear, and interactive effects of exposures on cardiovascular biomarkers.

The Markov Chain Monte Carlo (MCMC) algorithm was used for posterior sampling, employing a Gibbs sampler with 50,000 iterations. The first 10,000 iterations were discarded as burn-in to ensure the stability of posterior estimates. Convergence diagnostics were conducted to confirm the reliability of the results, including visual inspection of trace plots and the calculation of the Gelman–Rubin statistic. These diagnostics indicated satisfactory convergence of the MCMC chains.

The Bayesian Kernel Machine Regression approach is designed to analyze complex mixtures by modeling health outcomes as a function of multiple exposure components using a flexible kernel function [12]. This approach allows for the exploration of the joint and individual mixtures of pollutants such as arsenic, cadmium, and mercury on an outcome such as cardiovascular risk biomarkers. The BKMR model provided posterior inclusion probabilities (PIPs) to evaluate the contribution of various metal exposures to the cardiovascular outcomes examined in our study. These outcomes included systolic blood pressure, C-reactive protein, LDL cholesterol, HDL cholesterol, triglycerides, and total cholesterol. PIPs are a statistical measure that quantifies the likelihood of an exposure being a significant predictor while accounting for interactions among different exposures. This approach allows us to identify the relative importance of each metal in influencing these specific cardiovascular outcomes. All BKMR and regression analyses were adjusted for age, sex, and race/ethnicity to control for potential confounding effects.

### 2.6. Missing Data

Multiple Imputation by Chained Equations (MICE) was applied to handle missing data. This method ensured that missing values were imputed using all relevant variables, minimizing bias and maintaining data integrity. Imputation models incorporated sociodemographic characteristics, exposure levels, and cardiovascular outcomes to ensure consistency and reliability.

In this study, a small number of observations were missing across several variables. Systolic blood pressure each had one missing observation, while HDL cholesterol had 16 missing values. LDL cholesterol and total cholesterol were each missing for 17 participants, and triglycerides were missing for 15 participants. C-reactive protein had the highest missing values at 45, whereas arsenic and cadmium were missing for 21 and 32 participants, respectively. Finally, three participants were missing sex information. Age, mercury levels, and race/ethnicity had no missing data, providing a stable foundation for the imputation models.

Using the MICE approach, these missing values were imputed effectively, ensuring that analyses were not biased due to incomplete data while maintaining the integrity and robustness of the findings.

### 2.7. Statistical Significance and Software

For non-Bayesian analyses, a *p*-value of less than 0.05 was considered statistically significant. All statistical analyses were performed using R software (version 4.4.1), including specialized libraries for BKMR modeling, data visualization, and MICE imputation.

### 2.8. Ethical Considerations

The *All of Us* dataset was accessed under approved research guidelines, with participants providing informed consent. The *All of Us* research program adheres to rigorous ethical and data privacy standards to ensure participant confidentiality and data security. This study leveraged de-identified data. Thus, North Carolina Agricultural and Technical State University did not require IRB.

## 3. Results

### 3.1. Summary of Key Study Variables

In this study, we analyzed the cardiovascular biomarkers of 60 men and 73 women, with men having an average age of 58.2 and women with an average age of 55.6. Three participants did not list a sex. In this study, 104 individuals identified their race/ethnicity as White (mean age: 57.5 years), 10 as Black or African American (mean age: 63.2 years), 7 as Hispanic (mean age: 48.2), 3 as Asian (mean age: 49.7 years), and 12 as other race (mean age: 48.8 years).

Table 1 summarizes mean values for key heart health metrics and environmental exposures stratified by sex. Males had higher triglycerides (163 mg/dL), mercury (3.73 µg/L), and arsenic (9.96 µg/L) levels compared to females. Conversely, females show higher levels of C-reactive protein (CRP) (6.39 mg/L), low-density lipoprotein (LDL) (109 mg/dL), high-density lipoprotein (HDL) (63.1 mg/dL), and cadmium (0.595 µg/L).

Meanwhile, systolic blood pressure was slightly elevated in males. These findings highlight sex-based differences in lipid profiles, inflammatory markers, and environmental exposures, emphasizing the need for sex-specific analyses in cardiovascular and environmental health research.

### 3.2. Distribution of Heart Health Metrics by Race/Ethnicity

**Figure 1:**
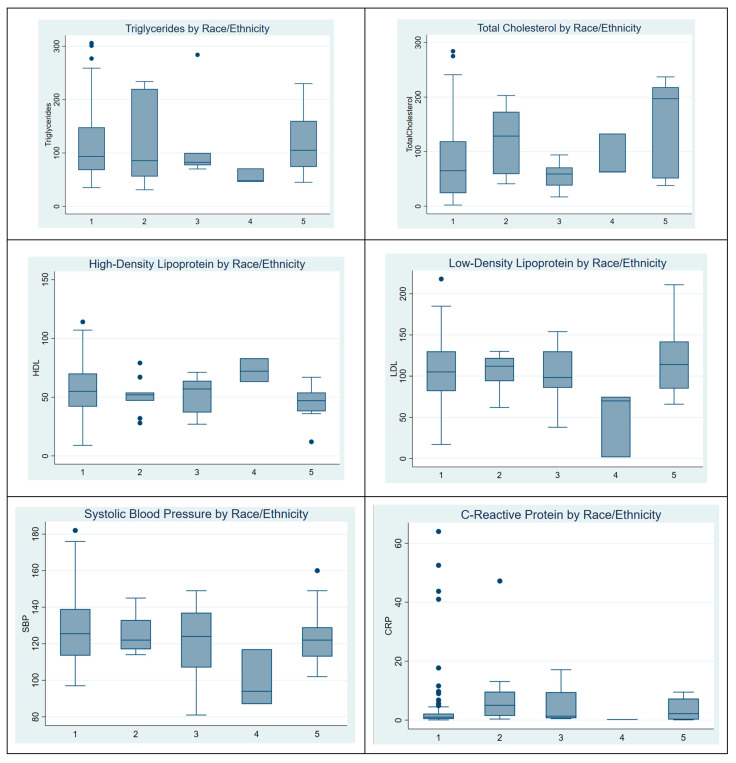
Distribution of heart health metrics by race/ethnicity.

Figure 1 shows the distribution of heart health metrics across race/ethnicity groups: White (1), Black (2), Hispanic (3), Asian (4), and Other (5). Notable patterns include Asian (4) groups displaying lower levels across most metrics, particularly for LDL and blood pressure (SBP). These findings suggest racial/ethnic differences in cardiovascular risk factors. This figure highlights the presence of outliers within different racial groups. Notably, there appears to be an outlier among White study participants. Additionally, C-reactive protein (CRP) levels exhibit a particularly high number of outliers across the dataset. Analysis of variance (ANOVA) was conducted to assess differences in cardiovascular-related biomarkers across race/ethnicity groups. Systolic blood pressure, C-reactive protein, HDL, LDL, triglycerides, and total cholesterol were evaluated. Significant differences were observed only for total cholesterol (*p* = 0.043).

### 3.3. Spearman Correlation Matrix of Heart Health Metrics and Environmental Exposures

**Figure 2:**
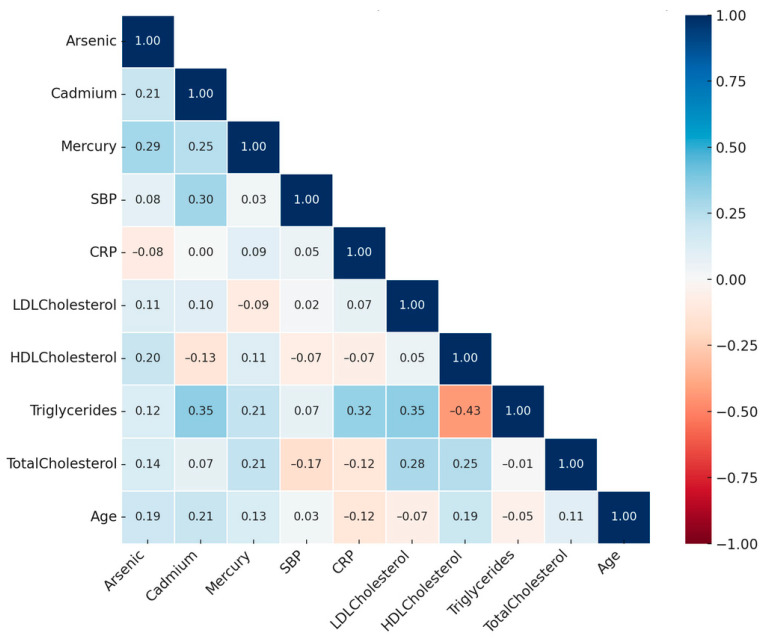
Spearman correlation matrix of heart health metrics and environmental exposures.

Figure 2 presents a Spearman correlation heatmap illustrating the pairwise relationships among cardiovascular health metrics and environmental exposures. The strength of correlations is visually represented, with darker red indicating strong positive associations and darker blue indicating strong negative associations. Following Cohen’s guidelines (1988) [14], correlations were classified as weak (|r| < 0.3), moderate (0.3 ≤ |r| < 0.5), and strong (|r| ≥ 0.5).

Systolic blood pressure (SBP) shows a moderate positive correlation with cadmium (|r| = 0.30), suggesting a potential association between blood pressure regulation and cadmium exposure. High-density lipoprotein cholesterol (HDL-C) exhibits a moderate inverse correlation with triglycerides (|r| = −0.43), consistent with their opposing roles in lipid transport and storage pathways. C-reactive protein (CRP), a marker of systemic inflammation, showed a weak positive correlation with mercury (|r| = 0.09) and a moderate correlation with triglycerides (|r| = 0.32).

Among environmental exposures, cadmium demonstrates a moderate positive correlation with triglycerides (|r| = 0.35) and a weak positive correlation with arsenic (|r| = 0.21), while mercury shows a weak positive correlation with arsenic (|r| = 0.29). These findings suggest shared pathways of exposure or accumulation across these metals.

These results highlight the relationships between cardiovascular biomarkers and environmental exposures as captured by the nonparametric Spearman correlation, providing a foundation to further explore their interactions and implications for cardiovascular health.

### 3.4. Regression Results for Key Cardiovascular Markers

In this study, we performed multivariable linear regression. Table 2 below presents the results of a linear regression analysis for various cardiovascular-related variables, including systolic blood pressure (SBP in mmHg), C-reactive protein (CRP in mg/L), low-density lipoprotein (LDL in mg/dL), high-density lipoprotein (HDL in mg/dL), triglycerides (in mg/dL), and total cholesterol (in mg/dL). Predictors for all analyses include arsenic, cadmium, and mercury (in µg/L), and the results are adjusted for age, race/ethnicity, and sex.

The results for SBP show that arsenic, cadmium, and mercury are not statistically significantly associated with SBP. However, examining specific trends, SBP increases with higher levels of arsenic and cadmium, while it decreases with higher levels of mercury.

The results for CRP indicate that arsenic, cadmium, and mercury are not statistically significantly associated with it. Nonetheless, CRP increases with higher levels of all three metals on average.

For LDL, none of the predictors (arsenic, cadmium, and mercury) are statistically significantly associated with LDL levels. However, the results suggest a trend where LDL increases as levels of arsenic, cadmium, and mercury increase.

Mercury was statistically significantly associated with HDL, while arsenic and cadmium were not. Specifically, HDL levels increase significantly with mercury levels and show a nonsignificant increase with arsenic. Conversely, HDL decreases with higher levels of cadmium.

For triglycerides, cadmium is significantly associated with triglycerides, while arsenic and mercury are not. Specifically, triglycerides significantly increase with cadmium levels, while they decrease (though not significantly) with increasing arsenic and mercury levels.

For total cholesterol, arsenic is significantly associated with it, whereas cadmium and mercury are not. As arsenic and mercury levels increase, total cholesterol levels increase (significantly for arsenic), while cadmium levels show a nonsignificant decreasing trend.

### 3.5. Bayesian Kernel Machine Regression Results

Within the Bayesian Kernel Machine Regression framework, this study evaluated the posterior inclusion probability, as well as univariate, bivariate, overall, single-variable, and single-variable interactive exposure–response effects. The outcome variables comprised cardiovascular-related measures, while the predictors included metals and metalloids. Covariates such as age, sex, and race/ethnicity were incorporated into the analysis. The results of these analyses are detailed in the subsequent sections.

#### 3.5.1. Posterior Inclusion Probability

Table 3 summarizes the posterior inclusion probability (PIP) values, which represent the probability that a specific environmental marker (arsenic, cadmium, or mercury) is included as a relevant predictor for a given health outcome, based on the Bayesian Kernel Machine Regression model. These values provide insights into the relative importance of each metal exposure in influencing various cardiovascular biomarkers. In this context, “relative importance” refers to the degree to which each metal contributes to explaining the variability in a specific cardiovascular biomarker compared to the other metals in the model.

The results indicate that mercury is the most relevant predictor for systolic blood pressure (SBP), HDL cholesterol, and triglycerides, as it has the highest PIP for these outcomes. For C-reactive protein (CRP), cadmium emerges as the most significant predictor. Arsenic has the highest PIP for low-density lipoprotein (LDL) cholesterol and total cholesterol, making it the most influential metal for these biomarkers. These findings highlight the varying levels of influence each metal has on specific cardiovascular health indicators and reinforce the importance of evaluating their combined effects.

#### 3.5.2. Univariate, Bivariate, Overall, Single-Variable, and Single-Variable Interactive Effects

**Figure 3:**
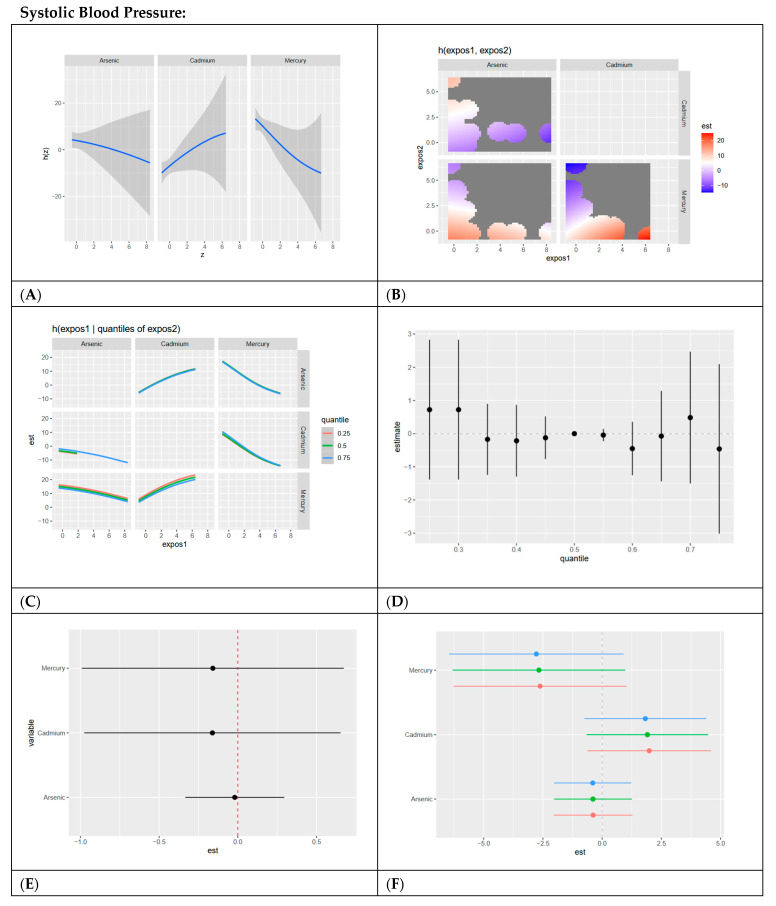
**BKMR results for systolic blood pressure.** (**A**) Univariate exposure–response functions with 95% credible intervals for the association between metal/metalloid exposures (arsenic, cadmium, and mercury) and systolic blood pressure, with other metal exposures fixed at the median. Analyses are adjusted for age, sex, ethnicity, and race. Results show a linear, slightly negative relationship for arsenic and mercury and a linear, positive relationship for cadmium. (**B**) Bivariate exposure–response functions illustrating the joint association of metal exposures with systolic blood pressure. Analyses are adjusted for age, sex, and race. Results show a strong joint association between high mercury and low cadmium levels, low mercury and arsenic across all exposure levels, and high cadmium and low mercury. (**C**) Bivariate exposure–response functions exploring the predictor–response relationships while varying quantiles (25th, 50th, 75th percentiles) of a second predictor, with all other exposures fixed. Analyses are adjusted for age, sex, and race. Results show a linear, slightly positive association between cadmium and other metals/metalloids at all exposure levels, with mercury and arsenic having slightly negative linear associations. (**D**) Summary of overall health effects and 95% credible intervals of combined metal/metalloid exposures on systolic blood pressure at various quantiles, ranging from the 25th to the 75th percentiles. Analyses are adjusted for age, sex, and race. Results show no clear pattern at increasing quantiles. (**E**) Single-variable interaction terms and 95% credible intervals for metals, comparing the effect of each metal exposure when all other metals are fixed at the 75th percentile compared to the 25th percentile. Analyses are adjusted for age, sex, and race. Results show minimal interaction between each metal/metalloid and combined exposure to other metals/metalloids. (**F**) Single-exposure attributable effects and 95% credible intervals, representing the change in systolic blood pressure associated with a change in a specific exposure (metal/metalloid) from its 25th to its 75th percentile, with all other exposures fixed at quantiles of 0.25 (red), 0.50 (green), or 0.75 (blue). Analyses are adjusted for age, sex, and race. Results show cadmium has the strongest positive effect, with minimal interaction demonstrated for all exposures as evidenced by red, green, and blue dots essentially overlapping.

**Figure 4:**
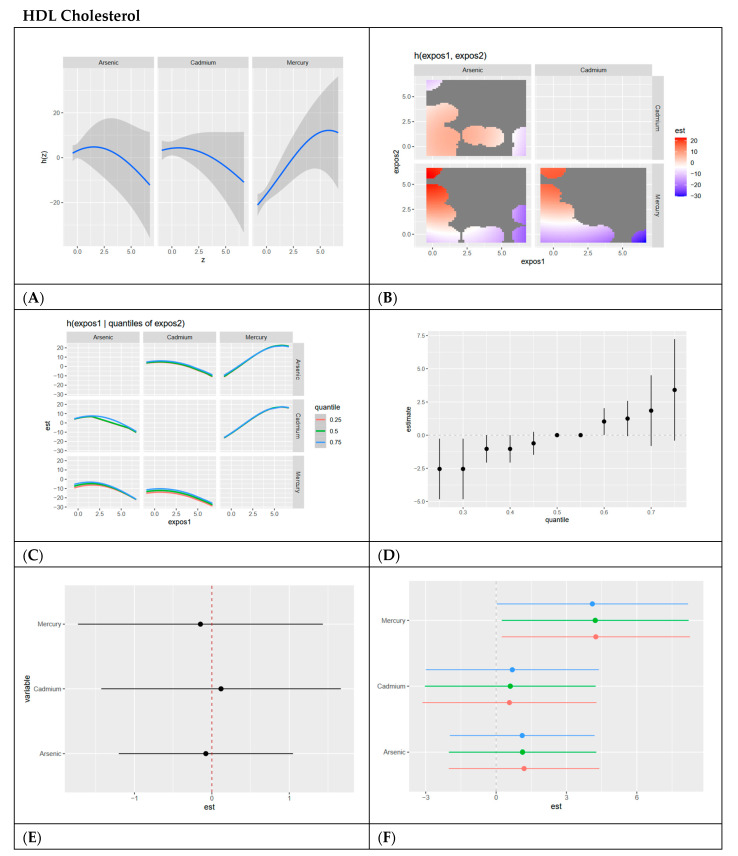
**BKMR results for HDL cholesterol.** (**A**) Univariate exposure–response functions with 95% credible intervals for the association between metal exposures (arsenic, cadmium, and mercury) and HDL cholesterol, with other metal exposures fixed at the median. Analyses are adjusted for age, sex, ethnicity, and race. Results show a slightly inverted U shapes for all three exposures with arsenic and cadmium decreasing as dose increases and mercury increasing as dose increases. (**B**) Bivariate exposure–response functions illustrating the joint association of metal exposures with HDL cholesterol. Analyses are adjusted for age, sex, and race. Results show a strong association between low arsenic and high mercury; arsenic, and mercury show a positive relationship across many exposure levels, with high mercury and low cadmium demonstrating strong associations. (**C**) Bivariate exposure–response functions exploring the predictor–response relationships while varying quantiles (25th, 50th, 75th percentiles) of a second predictor, with all other exposures fixed. Analyses are adjusted for age, sex, and race. Results show relatively flat semilinear curves for arsenic and cadmium with other exposures, with a positive linear curve for mercury with other exposures at the 0.75 quantile. (**D**) Summary of overall health effects and 95% credible intervals of metal exposures on HDL cholesterol at various quantiles, ranging from the 25th to the 75th percentiles. Analyses are adjusted for age, sex, and race. Results show a positive trend at increasing quantiles when exposed to all metals/metalloids. (**E**) Single-variable interaction and 95% credible intervals terms for metals, comparing the effect of each metal exposure when all other metals are fixed at the 75th percentile compared to the 25th percentile. Analyses are adjusted for age, sex, and race. Results show minimal interaction between each metal/metalloid and combined exposure to other metals/metalloids. (**F**) Single-exposure attributable effects and 95% credible intervals, representing the change in HDL cholesterol associated with a change in a specific exposure from its 25th to its 75th percentile, with all other exposures fixed at quantiles of 0.25 (red), 0.50 (green), or 0.75 (blue). Analyses are adjusted for age, sex, and race. Results show mercury has the most positive effect with minimal interaction for each exposure with other combined exposures, as demonstrated by red, green, and blue dots essentially overlapping.

**Figure 5:**
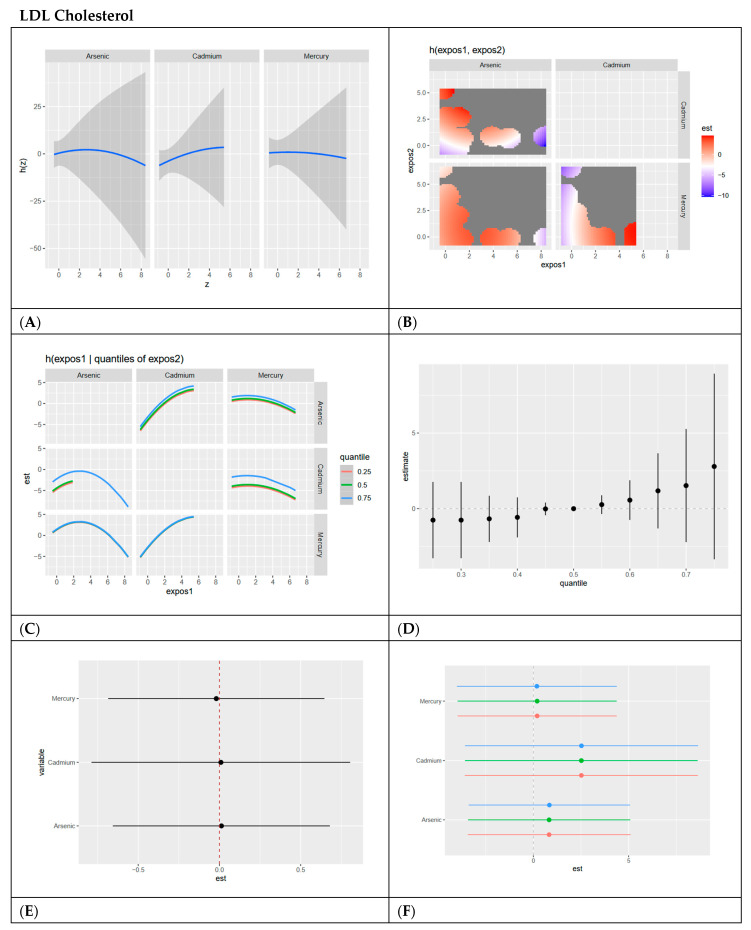
**BKMR results for LDL cholesterol.** (**A**) Univariate exposure–response functions with 95% credible intervals for the association between metal exposures (arsenic, cadmium, and mercury) and LDL cholesterol, with other metal exposures fixed at the median. Analyses are adjusted for age, sex, ethnicity, and race. Results show relatively flat curves for all exposures. (**B**) Bivariate exposure–response functions illustrating the joint association of metal exposures with LDL cholesterol. Analyses are adjusted for age, sex, and race. Results show a strong positive relationship between arsenic and cadmium at most exposure levels, with the strongest occurring at high cadmium and low arsenic. High arsenic and lower cadmium also show a strong negative relationship. (**C**) Bivariate exposure–response functions exploring the predictor–response relationships while varying quantiles (25th, 50th, 75th percentiles) of a second predictor, with all other exposures fixed. Analyses are adjusted for age, sex, and race. Results show an inverted U shape for arsenic and other metals, especially at the 0.75 quantile. For cadmium there is a positive relationship with other exposures as cadmium levels increase. Mercury has a relatively flat relationship with other exposures. (**D**) Summary of overall health effects and 95% credible intervals of metal exposures on LDL cholesterol at various quantiles, ranging from the 25th to the 75th percentiles. Analyses are adjusted for age, sex, and race. Results show that overall exposure has a slightly positive effect on LDL with more uncertainty at higher exposure levels as demonstrated by large credible interval. (**E**) Single-variable interaction and 95% credible intervals terms for metals, comparing the effect of each metal exposure when all other metals are fixed at the 75th percentile compared to the 25th percentile. Analyses are adjusted for age, sex, and race. Results demonstrate minimal interaction between each exposure with combined levels of other exposures as demonstrated by the dots all on or near the zero line. (**F**) Single-exposure attributable effects and 95% credible intervals, representing the change in LDL cholesterol associated with a change in a specific exposure from its 25th to its 75th percentile, with all other exposures fixed at quantiles of 0.25 (red), 0.50 (green), or 0.75 (blue). Analyses are adjusted for age, sex, and race. Results demonstrate cadmium has the most positive effect with minimal interaction for each exposure with other combined exposures as demonstrated by red, green and blue dots essentially overlapping.

**Figure 6:**
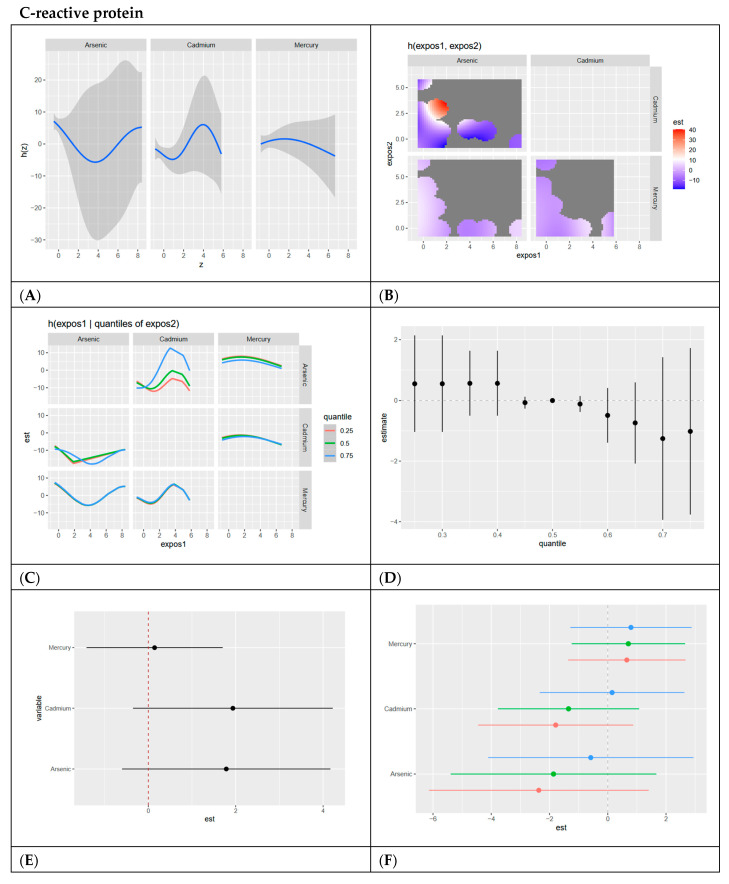
**BKMR results for C-reactive protein.** (**A**) Univariate exposure–response functions with 95% credible intervals for the association between metal exposures (arsenic, cadmium, and mercury) and CRP, with other metal exposures fixed at the median. Analyses are adjusted for age, sex, ethnicity, and race. Results indicate a clear U shape for arsenic, an inverted U shape for cadmium, and a relatively linear relationship for mercury. (**B**) Bivariate exposure–response functions illustrate the joint association of metal exposures with CRP. Analyses are adjusted for age, sex, and race. Results show negative relationships across all exposure levels, with the only positive relationship being at lower arsenic levels with medium cadmium levels. (**C**) Bivariate exposure–response functions exploring the predictor–response relationships while varying quantiles (25th, 50th, 75th percentiles) of a second predictor, with all other exposures fixed. Analyses are adjusted for age, sex, and race. Results indicate a clear U-shaped relationship between arsenic and mercury when mercury is at its 0.75 quantile. Arsenic shows a slightly less pronounced U-shaped relationship with cadmium when cadmium is at its 0.75 quantile and a V-shaped relationship with cadmium when cadmium is at its 0.25 and 0.5 quantile. (**D**) Summary of overall health effects and 95% credible intervals of metal exposures on CRP at various quantiles, ranging from the 25th to the 75th percentiles. Analyses are adjusted for age, sex, and race. Results indicate a slightly negative pattern with large, credible intervals at higher quantiles of exposure. (**E**) Single-variable interaction terms and 95% credible intervals for metals, comparing the effect of each metal exposure when all other metals are fixed at the 75th percentile compared to the 25th percentile. Analyses are adjusted for age, sex, and race. Results indicate an interaction between cadmium and arsenic exposure with combined levels of other exposures. (**F**) Single-exposure attributable effects and 95% credible intervals, representing the change in CRP associated with a change in a specific exposure from its 25th to its 75th percentile, with all other exposures fixed at quantiles of 0.25 (red), 0.50 (green), or 0.75 (blue). Analyses are adjusted for age, sex, and race. Results confirm the interaction of arsenic and cadmium with other exposures when the other exposures are fixed at different quantiles. The results also indicate mercury has the largest single-variable effect.

**Figure 7:**
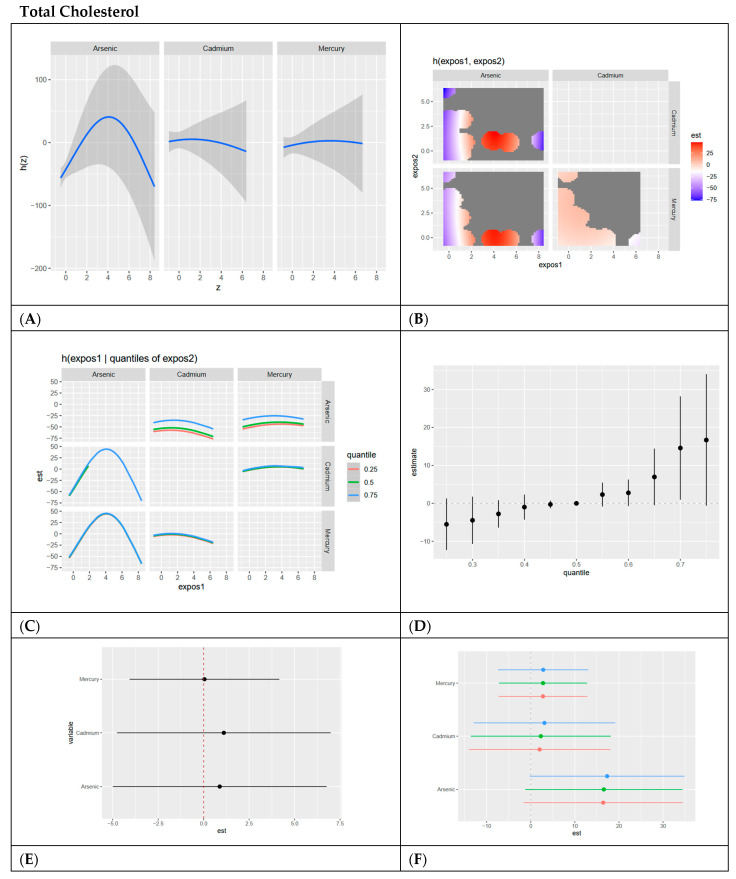
**BKMR results for total cholesterol.** (**A**) Univariate exposure–response functions with 95% credible intervals for the association between metal exposures (arsenic, cadmium, and mercury) and total cholesterol, with other metal exposures fixed at the median. Analyses are adjusted for age, sex, ethnicity, and race. Results indicate an inverted U-shaped relationship for arsenic and flat linear relationships for cadmium and mercury. (**B**) Bivariate exposure–response functions illustrating the joint association of metal exposures with total cholesterol. Analyses are adjusted for age, sex, and race. Results indicate a strong relationship between arsenic and cadmium at medium ranges for arsenic and lower levels for cadmium. Strong negative relationships exist at high levels of cadmium, low levels of arsenic, and high levels of arsenic and low levels of cadmium. (**C**) Bivariate exposure–response functions exploring the predictor–response relationships while varying quantiles (25th, 50th, and 75th percentiles) of a second predictor, with all other exposures fixed. Analyses are adjusted for age, sex, and race. Results indicate an inverted U-shaped relationship for Arsenic with cadmium and mercury, especially at the 0.75 quantile. Cadmium and mercury have flat relationships with other exposures at all visible quantiles of exposure. (**D**) Summary of overall health effects and 95% credible intervals of metal exposures on total cholesterol at various quantiles, ranging from the 25th to the 75th percentiles. Analyses are adjusted for age, sex, and race. Results indicate a strong positive relationship of all exposures together on total cholesterol and increasing quantiles, with credible intervals becoming larger as exposure increases. (**E**) Single-variable interaction terms and 95% credible intervals for metals, comparing the effect of each metal exposure when all other metals are fixed at the 75th percentile compared to the 25th percentile. Analyses are adjusted for age, sex, and race. Results indicate minimal interaction, with cadmium and arsenic showing only slight interaction. (**F**) Single-exposure attributable effects and 95% credible intervals, representing the change in total cholesterol associated with a change in a specific exposure from its 25th to its 75th percentile, with all other exposures fixed at quantiles of 0.25 (red), 0.50 (green), or 0.75 (blue). Analyses are adjusted for age, sex, and race. Results indicate arsenic has the largest positive single exposure effect and confirms minimal interaction between each metal/metalloid and combined levels of other metals/metalloids.

**Figure 8:**
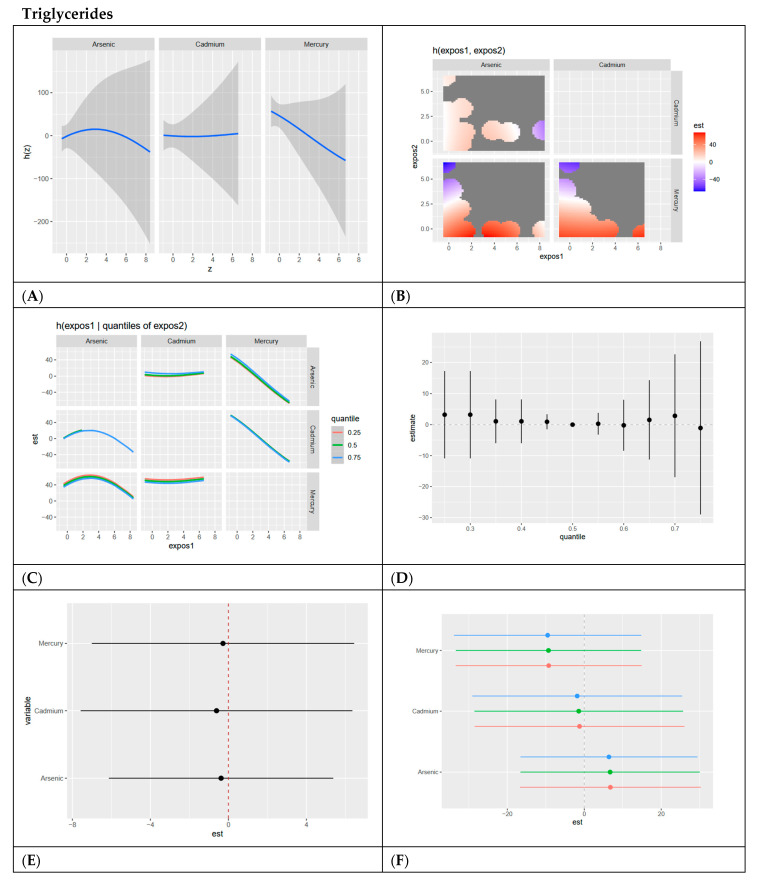
**BKMR results for triglycerides.** (**A**) Univariate exposure–response functions with 95% credible intervals for the association between metal exposures (arsenic, cadmium, and mercury) and triglycerides, with other metal exposures fixed at the median. Analyses are adjusted for age, sex, ethnicity, and race. Results show a slight inverted U shape for arsenic, a flat relationship for cadmium, and a negative relationship for mercury with triglycerides. (**B**) Bivariate exposure–response functions illustrating the joint association of metal exposures with triglycerides. Analyses are adjusted for age, sex, and race. Results generally show positive associations between combined metals with strong negative relationships existing for low arsenic and high mercury, and low cadmium and high mercury. (**C**) Bivariate exposure–response functions exploring the predictor–response relationships while varying quantiles (25th, 50th, 75th percentiles) of a second predictor, with all other exposures fixed. Analyses are adjusted for age, sex, and race. Results show that arsenic shows an inverted U shape with cadmium at 0.75 quantiles and mercury at all quantiles. Results also show that cadmium has a flat relationship with arsenic and mercury at all quantiles. Mercury has a negative relationship with arsenic at all quantiles and with cadmium at the 0.75 quantile. (**D**) Summary of overall health effects and 95% credible intervals of metal exposures on triglycerides at various quantiles, ranging from the 25th to the 75th percentiles. Analyses are adjusted for age, sex, and race. Results for the overall exposure effect show a flat relationship at all quantiles for the combined effect of metals/metalloids at increasing quantiles. (**E**) Single-variable interaction terms and 95% credible intervals for metals, comparing the effect of each metal exposure when all other metals are fixed at the 75th percentile compared to the 25th percentile. Analyses are adjusted for age, sex, and race. Results show minimal interaction. (**F**) Single-exposure attributable effects and 95% credible intervals, representing the change in triglycerides associated with a change in a specific exposure from its 25th to its 75th percentile, with all other exposures fixed at quantiles of 0.25 (red), 0.50 (green), or 0.75 (blue). Analyses are adjusted for age, sex, and race. Results confirm minimal interaction, with arsenic demonstrating the highest single variable effect.

In this section, we present BKMR analyses to examine the associations between metal/metalloid exposures (arsenic, cadmium, and mercury) and six cardiovascular-related biomarkers, including systolic blood pressure, HDL cholesterol, LDL cholesterol, CRP, total cholesterol, and triglycerides. For each biomarker, we provide a series of six visualizations (Figure 3, Figure 4, Figure 5, Figure 6, Figure 7 and Figure 8) that explore the univariate and bivariate exposure–response relationships, quantify interactions between exposures, and summarize the overall and quantile-specific effects of individual metals. These analyses, adjusted for key covariates such as age, sex, ethnicity, and race, allow for a comprehensive evaluation of both individual and joint effects of metal exposures on cardiovascular outcomes.

## 4. Discussion

### 4.1. Overview and Descriptive Statistics Results

In this study, we examined the associations between three metal exposures—arsenic, cadmium, and mercury—and six cardiovascular-related biomarkers: systolic blood pressure, HDL cholesterol, LDL cholesterol, C-reactive protein, total cholesterol, and triglycerides. We utilized both linear regression and Bayesian Kernel Machine Regression to identify significant relationships and unravel the complex, non-linear, and interactive effects of these exposures on cardiovascular health.

Men exhibited higher mean levels of blood pressure, triglycerides, mercury, and arsenic, whereas women had higher levels of CRP, LDL cholesterol, HDL cholesterol, total cholesterol, and cadmium. These observed sex differences may reflect variations in environmental exposure patterns, metabolism, or biological susceptibility to metal accumulation [15,16]. Such differences highlight the need to account for sex as a biological variable when assessing cardiovascular risks associated with environmental exposures.

### 4.2. Linear Regression Findings

Linear regression revealed significant positive associations between specific metals and cardiovascular biomarkers, providing initial insights into exposure–outcome relationships. For instance, mercury was positively associated with HDL cholesterol, a finding that might initially seem paradoxical given the established toxic effects of mercury. However, this result aligns with evidence suggesting that the primary source of mercury exposure in many populations—fish consumption—may influence lipid transport pathways positively. Fatty fish, such as salmon, mackerel, and sardines, are rich in omega-3 fatty acids, which are well documented for their cardioprotective effects. These include promoting HDL cholesterol (the “good” cholesterol), reducing triglycerides, improving endothelial function, and mitigating inflammation, which collectively enhance vascular health [17,18]. The observed positive association between mercury and HDL cholesterol may reflect the beneficial effects of omega-3 fatty acids outweighing the potential negative cardiovascular impacts of mercury exposure, especially in populations where fish constitutes a major dietary component [19,20]. However, this finding underscores the need for public health strategies that encourage the consumption of fish low in mercury (e.g., salmon and trout) to optimize cardiovascular benefits while minimizing toxic risks. Such guidance is particularly critical for populations that rely heavily on fish as a dietary staple or where mercury contamination in seafood is prevalent. Similarly, arsenic showed positive associations with triglycerides, while cadmium demonstrated a significant positive association with total cholesterol. Elevated triglycerides and total cholesterol are well-established risk factors for cardiovascular disease, including atherosclerosis, myocardial infarction, and stroke. These findings are consistent with prior research indicating that chronic arsenic exposure disrupts lipid metabolism, potentially through oxidative stress, mitochondrial dysfunction, and impaired lipid transport mechanisms [21,22]. Cadmium’s association with total cholesterol aligns with its known pro-inflammatory and endocrine-disrupting properties, which can adversely affect lipid homeostasis. Together, these results highlight the importance of considering not only individual metals but also their combined effects on lipid metabolism and cardiovascular health.

While these findings from linear regression are insightful, the inherent limitations of linear regression necessitated the application of Bayesian Kernel Machine Regression (BKMR). Linear regression assumes linearity, additivity, and independence among predictors, which may oversimplify the complex, real-world interactions between multiple exposures.

### 4.3. Posterior Inclusion Probabilities (PIPs)

Posterior inclusion probabilities (PIPs) are a Bayesian statistical measure derived from the BKMR analyses that represent the probability that a given exposure is included in the predictive model for the outcome, with higher PIP values indicating greater importance. Mercury was identified as the most influential exposure for SBP, HDL cholesterol, and triglycerides; cadmium for CRP; and arsenic for LDL cholesterol and total cholesterol. These PIP values reinforce the notion that the importance of specific metals varies by biomarker, highlighting the differential toxicological profiles of these exposures. For example, mercury’s prominence for HDL cholesterol may reflect its effect on oxidative stress pathways that influence cholesterol transport, while cadmium’s relevance for CRP aligns with its known inflammatory effects, although these exposures all contribute to cholesterol, inflammation, and hypertension [23].

### 4.4. BKMR Results and Exposure–Response Relationships

#### 4.4.1. Systolic Blood Pressure (SBP)

Univariate BKMR analyses revealed that cadmium had a positive association with SBP, while mercury demonstrated a negative association. This suggests that cadmium exposure may directly contribute to elevated SBP, consistent with its vascular toxicity [24], whereas mercury’s effect may be due to cardioprotective factors related to diet. Bivariate analyses confirmed these trends, showing positive interactions between cadmium and other metals. However, the overall exposure effect of all metals on SBP across quantiles showed no clear pattern, indicating potential variability in the combined effects of metal mixtures. The single-variable analysis identified cadmium as the dominant contributor, with minimal interaction effects.

#### 4.4.2. HDL Cholesterol

Univariate analyses showed a positive relationship for mercury with HDL cholesterol, while arsenic and cadmium displayed negative trends. The bivariate heatmap revealed complex interactions, with combinations of high mercury and low arsenic or cadmium showing strong positive effects on HDL cholesterol. Conversely, high cadmium and low mercury had strong negative effects, indicating the strong role of cadmium in cardiovascular dysfunction. These findings suggest that cadmium may play a unique role in lipid transport regulation, possibly through its impact on cholesterol efflux pathways. Finally, in our analysis, the overall exposure effect was positive, with mercury having the largest single-variable effect and minimal evidence of interaction effects.

Interestingly, mercury’s associations with HDL cholesterol and its prominence in PIPs for triglycerides and SBP may reflect its primary source—fish consumption. Fish, particularly fatty fish, are rich in omega-3 fatty acids, which have well-established protective cardiovascular effects, including promoting HDL cholesterol, reducing triglycerides, and improving vascular function. The observed positive associations between mercury and HDL cholesterol may therefore be driven more by the beneficial effects of omega-3 fatty acids than by mercury toxicity. This balance suggests that the cardioprotective effects of omega-3 fatty acids may outweigh the negative impacts of mercury exposure, particularly in populations where fish is a primary dietary source of mercury. Public health recommendations should emphasize the consumption of fish low in mercury to optimize cardiovascular benefits while minimizing toxic risks [25].

#### 4.4.3. LDL Cholesterol

The univariate exposure–response functions for LDL cholesterol were largely flat, indicating weak individual associations for arsenic, cadmium, and mercury. However, bivariate analyses revealed stronger interactions between arsenic and cadmium, particularly at higher exposure quantiles. The inverse U-shaped relationship observed for arsenic suggests that moderate arsenic exposure may dysregulate lipid synthesis and clearance, while higher levels may trigger compensatory mechanisms that normalize LDL cholesterol [26]. Cadmium’s positive association with arsenic and mercury in the bivariate analysis indicates a strong combined effect. The overall exposure effect showed a positive trend with widening credible intervals at higher quantiles, reflecting increased variability in response to combined exposures.

#### 4.4.4. C-Reactive Protein (CRP)

Univariate analyses revealed a U-shaped relationship for arsenic and an inverted U-shaped relationship for cadmium with CRP. The U shape for arsenic suggests that both low and high levels of exposure may trigger inflammation, possibly through oxidative stress and immune dysregulation. Similarly, the inverted U shape for cadmium for cadmium may reflect complex dose-dependent effects, where moderate exposures exacerbate inflammation, but very high levels may suppress inflammatory pathways [27]. Bivariate analyses showed strong interactions, particularly at higher quantiles of cadmium and mercury with arsenic. The overall exposure effect demonstrated considerable variability, with large credible intervals at extreme exposure quantiles, highlighting the complex and non-linear nature of these interactions.

#### 4.4.5. Total Cholesterol

Arsenic displayed an inverse U-shaped relationship with total cholesterol, indicating that moderate arsenic exposure may disrupt lipid metabolism, while higher levels potentially activate compensatory mechanisms [28]. Bivariate analyses confirmed these trends, with arsenic showing the highest effects when interacting with cadmium and mercury at moderate exposure levels. The overall exposure effect showed a positive trend, with arsenic identified as the largest single-variable contributor. Notably, arsenic and cadmium demonstrated significant interaction effects, emphasizing the need to consider combined exposures when evaluating total cholesterol.

#### 4.4.6. Triglycerides

For triglycerides, univariate analyses showed minimal effects for arsenic and cadmium, while mercury had a negative association. The bivariate heatmap indicated generally positive interactions between metals, although mercury’s negative relationship persisted. Quantile-based bivariate analyses revealed flat relationships for arsenic and cadmium but a consistent negative effect for mercury. These findings suggest that mercury may play a role in triglyceride metabolism, potentially through its influence on hepatic lipid processing [29]. The overall exposure effect was flat, with arsenic showing the largest single-variable effect and minimal interaction effects.

### 4.5. Strengths and Limitations

A key strength of this study is the use of BKMR to model non-linear and interactive effects of metal mixtures, providing a comprehensive understanding of exposure–response relationships. The sample size, though sufficient for detecting general trends, may limit the statistical power to detect subtle effects or interactions, particularly at extreme exposure levels or for subgroups. Missing data, while addressed using Multiple Imputation by Chained Equations, may still introduce bias if the missingness mechanism is not fully random. Additionally, unmeasured confounders, such as dietary patterns, physical activity, and other lifestyle factors, could influence the observed associations. While the study adjusted for age, sex, race, and ethnicity, these adjustments do not entirely account for the complexity of potential confounding factors. The cross-sectional design limits causal inference, and unmeasured confounding may influence the results. Future studies should adopt longitudinal designs to better capture temporal relationships and underlying mechanisms.

## 5. Conclusions

This study highlights the associations between arsenic, cadmium, and mercury exposures and cardiovascular disease (CVD) indicators. Mercury exposure was positively associated with HDL cholesterol but negatively associated with triglycerides and systolic blood pressure, suggesting complex relationships influenced by dietary sources like fish consumption. Arsenic exposure demonstrated positive associations with total cholesterol and triglycerides, indicating its potential role in dysregulating lipid metabolism. Cadmium exposure was positively associated with C-reactive protein and triglycerides, aligning with its known pro-inflammatory effects.

Bayesian Kernel Machine Regression (BKMR) revealed significant non-linear and interactive effects, with mercury emerging as the most influential predictor for HDL cholesterol, triglycerides, and systolic blood pressure; cadmium for C-reactive protein; and arsenic for LDL cholesterol and total cholesterol. Combined effects analyses showed that interactions between metals, particularly arsenic and cadmium, influence certain CVD indicators, underscoring the need for mixture-based approaches in environmental health research. These findings emphasize the importance of evaluating both individual and combined effects of metal exposures on cardiovascular health and call for further longitudinal studies to elucidate underlying mechanisms.

## Figures and Tables

**Table 1:** Mean levels of heart health metrics and environmental exposures by sex (m = overall missing data points across all variables; n indicates sample size).

Variable	Male (*n* = 60)	Female (*n* = 73)	Total (*n* = 133)	P (>|t|)
Age	58.2	55.6	56.8	0.330
Systolic blood pressure (SBP) mm[Hg]	127	125	126	0.5089
C-reactive protein (CRP) mg/L	3.54	6.39	5.19	0.233
Low-density lipoprotein (LDL) mg/dL	99.5	109	105	0.197
High-density lipoprotein (HDL) mg/dL	47.0	63.1	56.3	<0.001
Triglycerides mg/dL	163	105	130	0.061
Total cholesterol mg/dL	87.1	94.6	91.4	0.583
Cadmium μg/L	0.505	0.595	0.555	0.367
Mercury μg/L	3.73	3.39	3.55	0.661
Arsenic μg/L	9.96	6.20	7.91	0.262

**Table 2:** Linear regression results for metals with cardiovascular-related variables.

Outcome	Variable	+Estimate	Std. Error	t-Value	*p*-Value
Systolic Blood Pressure (SBP)	Arsenic	0.243	0.315	0.773	0.445
	Cadmium	6.70	4.48	1.49	0.144
	Mercury	−0.118	0.586	−0.201	0.842
C-Reactive Protein (CRP)	Arsenic	0.217	0.181	1.20	0.238
	Cadmium	2.99	2.58	1.16	0.253
	Mercury	0.089	0.337	0.264	0.793
Low-Density Lipoprotein (LDL)	Arsenic	0.249	0.510	0.488	0.629
	Cadmium	6.15	7.26	0.847	0.402
	Mercury	0.923	0.949	0.973	0.337
High-Density Lipoprotein (HDL)	Arsenic	0.383	0.272	1.41	0.168
	Cadmium	−5.39	3.87	−1.40	0.172
	Mercury	1.41	0.506	2.79	0.008 **
Triglycerides	Arsenic	−0.450	0.800	−0.563	0.577
	Cadmium	35.9	11.4	3.152	0.0321 **
	Mercury	−2.66	1.49	−1.79	0.0821
Total Cholesterol	Arsenic	2.67	0.917	2.91	0.00602 **
	Cadmium	−7.51	13.0	−0.576	0.568
	Mercury	1.67	1.70	0.978	0.334

Adjusted for age, race, and sex; ** statistically significant, *p* < 0.05.

**Table 3:** Posterior inclusion probability (PIP) for environmental markers by outcome.

Outcome	Variable	PIP
Systolic Blood Pressure	Arsenic	0.0302
	Cadmium	0.222
	Mercury	0.399
HDL Cholesterol	Arsenic	0.581
	Cadmium	0.332
	Mercury	0.980
LDL Cholesterol	Arsenic	0.108
	Cadmium	0.101
	Mercury	0.0726
C-reactive protein (CRP)	Arsenic	0.654
	Cadmium	0.734
	Mercury	0.349
Total Cholesterol	Arsenic	0.811
	Cadmium	0.276
	Mercury	0.242
Triglycerides	Arsenic	0.278
	Cadmium	0.214
	Mercury	0.353

## Data Availability

Data used in this study are available as a featured workspace to registered researchers of the All of Us researcher workbench. For information about access, please visit https://www.researchallofus.org/ (accessed on 7 January 2025).
